# No-Show Rates in a Cardiology Clinic During the COVID-19 Pandemic: A Retrospective Analysis From a Safety-Net Hospital

**DOI:** 10.7759/cureus.80897

**Published:** 2025-03-20

**Authors:** Khalid Sawalha, Andrew J Fancher, Subhi Al'Aref, Angel Lopez Candales

**Affiliations:** 1 Cardiovascular Disease, University of Arkansas for Medical Sciences, Little Rock, USA; 2 Internal Medicine, University of Kansas School of Medicine-Wichita, Wichita, USA; 3 Cardiology, University of Arkansas for Medical Sciences, Little Rock, USA; 4 Cardiovascular Medicine, University of Missouri Kansas City, Kansas City, USA

**Keywords:** cardiology outpatient clinic, covid-19 impact on cardiovascular outpatient, covid-19 pandemic, no show, safety net hospital

## Abstract

Background: The COVID-19 pandemic proved to be a formidable crisis leading to massive disruptions in healthcare delivery that compromised routine access to care across the United States. The strain of the pandemic on the healthcare system limited routine outpatient visits and regular follow-up for all patients. Patients struggling with social determinants of health (SDOH) have long suffered from limited access to healthcare and the pandemic exacerbated this issue. Patients with chronic cardiac conditions need regular follow-up to ensure the highest quality of care and limited outpatient accessibility can be highly disruptive to their health. Thus, patients with chronic cardiac conditions who also struggle with SDOH were a population that was particularly vulnerable to the strain of the pandemic.

Methods: A retrospective analysis was performed to assess the impact of the COVID-19 pandemic on the urban underserved patient population. Data were collected from the University Health System in Kansas City, MO, between 2019 and 2022. This safety-net hospital was selected because 66% of its patients are on Medicare, Medicaid, or uninsured, making its patient population a strong representation of the larger U.S. population struggling with SDOH. A report of in-person, outpatient cardiology visits scheduled during the specified timeframe was generated with demographic data and visit status. All visits scheduled that were completed or resulted in a no-show were included, with all other visits excluded. No-show rates were calculated overall and within each subgroup analyzed by dividing the number of no-shows by the total number of visits scheduled. No-show rates and percent change in no-show rates by year were analyzed for the whole population and stratified by gender, race, ethnicity, and insurance status.

Results: No-show rates increased in 12 out of 17 (71.0%) patient subgroups including male patients, Black patients, non-Hispanic patients, patients in the “other” category of ethnicity, those on a Self-Pay Discount Program, Medicaid, and Medicaid MC Plus. These groups all had no-show rates suggesting that nearly or greater than a third of their patients were not receiving the cardiovascular care they needed by the end of the pandemic. The highest no-show rates in 2022 were observed in the following patient subgroups: Black 1,915 (35.08%), Self-Pay Discount 490 (39.3%), Medicaid 873 (36.2%), and Medicaid MC Plus 689 (32.9%).

Conclusion: The COVID-19 pandemic significantly increased no-show rates for outpatient cardiology visits, particularly among underserved populations, highlighting the vulnerability of low socioeconomic status patients. This disruption in routine care underscores the need for continued development of strategies to ensure consistent healthcare access during crises. University Health System employs many outreach and other programs to help those struggling with SDOH and yet the strain of the pandemic was still seen. Other studies have demonstrated that telehealth may serve as a bridge to addressing care gaps in the underserved population. Further research is required to assess the short and long-term health impacts of missed appointments and to continue developing solutions for improving healthcare access in patients struggling with SDOH.

## Introduction

The COVID-19 pandemic was a global crisis that had a massive impact on the economy, healthcare systems, and daily lives of people worldwide. High-quality cardiovascular care relies on early detection and swift intervention to prevent progression of disease, and regular follow-up of patients with chronic conditions. All aspects of care were affected by the strain the pandemic placed on healthcare systems, and we are still trying to understand the full extent of the consequences. Many questions as to how the pandemic strain on the healthcare system affected access to care and patient outcomes have not yet been answered. Our study sought to investigate how the pandemic affected delivery of outpatient cardiovascular care, particularly in the underserved population who already struggled with access to care.

The inpatient aspects of cardiovascular care were affected by a shift in focus of hospital resources. Hospitals worldwide were overwhelmed with COVID-19 patients due to resource scarcity. This forced them to develop and implement newly created risk-stratification algorithms that prioritized COVID-19 patients which ultimately resulted in a worldwide decrease in inpatient cardiac patient inflow [1]. The newly developed policies led to mass cancellations of non-emergent interventions and testing essentially eliminating elective and routine inpatient procedures, but this also affected non-emergent admissions [1]. During the pandemic years, a decrease in hospital presentation for ischemic heart disease, decompensated heart failure, and endocarditis among other cardiac conditions was observed globally [1]. This was in part because many hospitals were also left understaffed and forced to prioritize emergent inpatient evaluations for non-COVID-related diseases [1,2]. While important at the time to help channel resources to pandemic-related efforts, these new hospital policies lead to a significant reduction in inpatient cardiovascular care. In the outpatient cardiovascular world, this and the direct effects of the pandemic manifested in a dramatic decrease in outpatient consultations, routine evaluations, and testing. Reports on trends of cardiac testing including electrocardiograms (EKGs), echocardiograms, outpatient monitors, stress testing, and cardiac computed tomography angiography (CCTA) frequently cite decreases of 50% or more during the initial course of the pandemic [1,3,4]. The effects of the strain placed on inpatient cardiovascular care are concerning, but the effects on outpatient care are even more dramatic.

Outpatient care and regular follow-up are critical components of high-quality cardiovascular care that unfortunately suffered greatly during the pandemic. Many chronic cardiac conditions benefit from regular follow-up whether simply for monitoring or for active adjustments to management plans. Heart failure is a good example of a condition significantly affected by disruption in outpatient care. A patient with newly discovered heart failure, once stabilized inpatient, needs workup to elucidate the cause of their heart failure that is not always completed inpatient. They also need to be started on guideline-directed medical therapy (GDMT) that requires careful addition of medications from each of the four pillars and up-titration of dosing to reach target doses at which the maximum benefits have been observed. Without regular follow-up, these aspects of heart failure management cannot be achieved.

For all cardiovascular patients, care suffered as outpatient visits declined. Some networks saw mild decreases in overall outpatient cardiology visits of 20-35% [3,5]. However, many saw more dramatic decreases with some investigations reporting an 80-93% decrease in outpatient visits [1,4]. This stems from well-intended messaging to the public to avoid hospitals and seek telemedicine appointments, when possible, in combination with global shutdowns and limitations in healthcare staffing [1,6]. But ultimately, this likely played some role in the reduction in outpatient visits. Similar effects were seen in primary care settings and a decrease in cardiology referrals was observed with one study showing an 80% decrease in outpatient cardiology referrals [4]. Patients of low socioeconomic status have long struggled with access to medical care and represent a group that was particularly vulnerable to the global effects of decreased access to standard medical care during the pandemic. University Health System in Kansas City, MO, serves a primarily urban and underserved population with 66% of its patients on Medicare, Medicaid, or uninsured. Our project sought to investigate the effects of the pandemic on access to outpatient cardiovascular care in this safety-net hospital system.

## Materials and methods

Study design

To assess the impact of the COVID-19 pandemic on outpatient cardiovascular care in underserved populations, data from outpatient cardiology clinics within the University Health System in Kansas City, Missouri, from 2019 to 2022 were retrospectively analyzed. Data were collected in 2023 and encompasses data from before the pandemic through 2022. The clinics employ electronic medical records (EMRs) which routinely track patient demographics when appointment was scheduled, appointment status as it changes during the visit, appointment date and type, check-in date and time, cycle time (from check-in to check-out), and insurance status, among other data. For this study, a report was generated with all details routinely collected by these EMRs (excluding unique patient identifiers other than medical record number {MRN}) for visits with appointment status “checked out” or “no-show” with other appointment statuses excluded. This was intended to isolate all completed visits and visits in which the appointment was scheduled, and provider was available, but the patient did not check in after 15 minutes from his scheduled appointment time, colloquially described as a “no-show.” Visits that were canceled or rescheduled prior to the time of appointment were not included in our dataset as we had no feasible way of assessing what caused rescheduling or cancellation. In contrast, in the case of a "no-show," we can reasonably conclude that both the patient and the provider intended to meet at the agreed-upon time, but the patient was unable to attend due to unforeseen circumstances. To ensure patient confidentiality, unique patient identifiers collected were minimized by pulling only MRN to specify each unique visit in the initial report. After MRN was used to remove duplicates for specific datasets as noted in our description of statistical analysis, this information was destroyed from the dataset prior to further analysis.

Study population and sample size

Our study population consisted of all patients scheduled for outpatient cardiology appointments in the specified time frame within the University Health System in Kansas City. The University Health System is a critical safety net healthcare system within the larger Kansas City metro area. Their central mission is to provide care to the medically underserved. University Health provides approximately $150,000,000 in uncompensated care yearly with nearly 66% of their patients on Medicare, Medicaid, or without insurance. For this reason, patients within this system represent an excellent sample of the larger underserved population. We chose to include all patients who were scheduled and either completed their visit or were not shown within the cardiology clinics to provide the best full-spectrum view of how the pandemic affected outpatient cardiovascular care.

Study measures

Data were collected on patient gender, race, ethnicity, and insurance status. Gender was separated into male and female patients. Race was separated into White, Black, and multiple other subgroups including Asian, Native American, “multiple,” “not listed,” and “patient declined.” However, the remaining subgroups were much smaller in proportion compared to the White and Black populations and were combined into one “other” group to facilitate statistical analysis. Similarly, ethnicity was separated into non-Hispanic, Hispanic, and multiple subgroups including “multiple,” “not listed,” and “patient declined.” These additional subgroups were also significantly small in proportion to the first two subgroups and did not provide much additional information; thus, to facilitate statistical analysis they were combined into one “other” group. Insurance status was separated into Self-Pay Discount (a payment plan for those who are uninsured), Medicare, Medicaid, Medicare replacement, Medicaid MC Plus, Blue Cross, United Healthcare, Aetna, and all others or not listed entries were combined into one “other” category.

For each year and total timespan, the number of patients, number of visits scheduled, and number of no-shows were counted. For each year and total timespan, these counts were repeated among each subgroup. The primary outcome measure was the no-show rate with secondary measures of difference in no-show rate and percent change in no-show rate compared to the previous year and between 2022 and 2019. This analysis was also completed for each subgroup. All unique visits contained data on completion or no-show status. All visits also had data present on patient gender and age. The only missing data occurred for self-reported information in the insurance status, race, and ethnicity categories which was processed as described above.

Statistical analysis

A retrospective analysis was performed on this data to compare no-show rates within different patient sub-groups for each year and to compare trends in no-show rates throughout the pandemic and between groups. To collect demographic data by number of patients, a second copy of the full data file, which included multiple visits for the same patients, was created. Duplicate entries were removed by eliminating duplicate MRNs resulting in two collections of data - one with information on every unique patient seen within each year and one with each unique visit that was scheduled within each year. Additionally, a third dataset was created combining data from all four years with duplicates removed by MRN to create a dataset of all unique patients seen in the University Health cardiology clinics within the selected four years.

The first data set was used to count the number of total patients and the number of patients within multiple subgroups for each year. Within each year, the count of each subgroup was divided by the total number of patients for that year to calculate what percent of the year’s population that subgroup represented. Additionally, mean age and standard deviation were calculated for each year. This process was repeated in the third dataset to count the number of unique patients within each subgroup and what percent of the total population this represented where the total population consisted of every unique patient seen by the clinics over all four years. The mean age and standard deviation for all unique patients seen in the clinic over four years were also calculated. A similar process was used in the second data set; however, in this dataset, every entry represented a unique visit rather than a unique patient. The total number of visits and “no-shows” was counted in each year and within each subgroup. No-show rate was calculated by dividing the number of no-shows by the total visits for the total population and each subgroup within each year. Differences in no-show rate between subgroups were tested for statistical significance using a two-sample z-test and chi-squared analysis. In the chi-squared analysis, the expected frequency was calculated using the no-show rate for the whole population within that year multiplied by the number of scheduled visits for the subgroup. Data from all subgroups met assumptions for chi-squared analysis. Additionally, no-show rates were compared over time by calculating the percent change in no-show rates from the year previous, excluding 2019 for which we did not have data from the previous year to compare. Percent change in no-show rate was also calculated between 2019 and 2022 to represent the change over the whole period studied. Percent change in no-show rate was chosen to compare trends in no-show rate over time in a time-series analysis.

Ethics statement

This study performed a retrospective analysis of data previously collected by the outpatient cardiology clinics. This study was exempt from a full institutional review board (IRB) review as it involved secondary analysis of anonymized data. It was conducted in accordance with the Declaration of Helsinki principles for medical research involving archived records. Patient identifiers, other than the unique MRN, were not collected in the initial report, and these were destroyed at the earliest opportunity, as detailed above. Although the study was exempt from IRB oversight, it was conducted in accordance with guidelines set out by the University of Missouri-Kansas City (UMKC) IRB to protect patient confidentiality. Best practices for protecting patient confidentiality in data storage and access were observed. The research team has no conflicts of interest to disclose.

## Results

Data were collected from outpatient cardiology clinics within the University Health Center System in Kansas City, MO, on all visits from 2019 to 2022. As noted above, data from all visits that were completed or resulted in a no-show were compiled for retrospective analysis. From January 1, 2019, to December 31, 2022, 12,976 patients were scheduled for 45,817 visits. The patients had a mean age of 54.3±15 years. Of this total population, 5,984 (46.12%) were male and 6,992 (53.88%) were female. A total of 6,057 (46.68%) patients were White, 5,068 (39.06%) were Black, and the remaining 1,851 (14.26%) patients were classified as "other." Among the patients, 11,427 (88.06%) self-identified as non-Hispanic, 873 (6.73%) identified as Hispanic, and the remaining 676 (5.21%) were categorized as "other." The distribution of insurance status was as follows: 1,283 (9.88%) were on a Self-Pay Discount plan, 1,179 (9.08%) had Medicare, 1,437 (11.07%) had Medicaid, 2,409 (18.57%) had Medicare replacement, 2,346 (18.08%) had Medicaid MC Plus, 1,275 (9.83%) had Blue Cross, 238 (1.83%) had United Healthcare, 187 (1.44%) had Aetna, and the remaining 803 (6.19%) were in the "other" category. This data, in addition to demographic data by year, are displayed in Table 1.

**Table 1:** Demographic information for each year and total population, including patient count, visits, no-shows, mean age (with standard deviation), and distribution by subcategory (percent of total population).

Variables	2019	2020	2021	2022	2019-2022
Patients	5,180	4,876	5,456	6,017	12,976
Visits	11,279	9,990	11,874	12,674	45,817
No-shows (%)	3,168 (28.1)	2,606 (26.1)	3,360 (28.3)	3,878 (30.6)	13,012 (28.4)
Mean age (years)	55.8±14	55.5±14	55.6±14	55.8±14	54.3±15
Gender					
Male (%)	2,501 (48.3)	2,382 (48.9)	2,566 (47.0)	2,847 (47.3)	5,984 (46.12)
Female (%)	2,679 (51.7)	2,494 (51.1)	2,890 (53.0)	3,170 (52.7)	6,992 (53.88)
Race					
White (%)	2,434 (47.0)	2,296 (47.1)	2,497 (45.8)	2,716 (45.1)	6,057 (46.68)
Black (%)	2,079 (40.1)	1,933 (39.6)	2,219 (40.7)	2,484 (41.3)	5,068 (39.06)
Other (%)	667 (12.9)	647 (13.3)	740 (13.6)	817 (13.6)	1,851 (14.26)
Ethnicity					
Non-Hispanic (%)	4,529 (87.4)	4,338 (89.0)	4,906 (89.9)	5,318 (88.4)	11,427 (88.06)
Hispanic (%)	255 (4.92)	292 (5.99)	353 (6.47)	438 (7.28)	873 (6.73)
Other (%)	396 (7.65)	246 (5.05)	197 (3.61)	261 (4.34)	676 (5.21)
Insurance status					
Self-Pay Discount (%)	1,235 (23.8)	1,145 (23.5)	1,244 (22.8)	757 (12.6)	1,283 (9.887)
Medicare (%)	874 (16.9)	663 (13.6)	672 (12.3)	585 (9.72)	1,179 (9.086)
Medicaid (%)	1,020 (19.7)	980 (20.1)	985 (18.1)	1,105 (18.4)	1,437 (11.07)
Medicare replacement (%)	863 (16.7)	867 (17.8)	1,044 (19.1)	1,306 (21.7)	2,409 (18.57)
Medicaid MC Plus (%)	151 (2.92)	178 (3.65)	302 (5.54)	912 (15.2)	2,346 (18.08)
Blue Cross (%)	622 (12.0)	625 (12.8)	731 (13.4)	802 (13.3)	1,275 (9.83)
United Healthcare (%)	68 (1.31)	74 (1.52)	98 (1.80)	119 (1.98)	238 (1.83)
Aetna (%)	59 (1.14)	70 (1.44)	69 (1.26)	89 (1.48)	187 (1.44)
Other (%)	288 (5.56)	274 (5.62)	311 (5.70)	342 (5.68)	803 (6.19)

The no-show rate was calculated for the total population and each subgroup to compare differences and analyze trends over time. In 2019, a total of 5,180 patients were scheduled for 11,279 visits, with 3,168 (28.09%) no-shows. Of these, 2,501 (48.3%) were male patients scheduled for 5,715 visits, with 1,654 (28.94%) no-shows. A total of 2,679 (51.7%) were female patients scheduled for 5,564 visits, with 1,514 (27.21%) no-shows. The difference between male and female no-show rates was 1.73 percentage points, statistically significant with a Z-statistic of 2.04 (p<0.04). The effect size, measured by Cohen's d, was 0.04, indicating a small effect. Among racial subgroups, 2,434 (47.0%) patients were White, with 5,049 visits scheduled and 1,195 (23.67%) no-shows. A total of 2,079 (40.1%) patients were Black, with 4,932 visits scheduled and 1,568 (31.79%) no-shows. The remaining 667 (12.9%) patients were categorized as "other," with 1,298 visits scheduled and 405 (31.2%) no-shows. The differences in no-show rates among these subgroups were statistically significant by chi-squared analysis (chi-squared = 63.69, p<0.001), with Cramer’s V of 0.08, indicating negligible association. In terms of ethnicity, 4,529 (87.4%) patients identified as non-Hispanic, with 10,057 visits scheduled and 2,792 (27.76%) no-shows. A total of 255 (4.92%) patients identified as Hispanic, with 519 visits scheduled and 152 (29.3%) no-shows. A total of 396 (7.65%) patients fell into the "other" category, with 703 visits scheduled and 224 (31.9%) no-shows. The differences in no-show rates among these subgroups were not statistically significant (chi-squared = 4.21, p>0.05), with Cramer’s V of 0.02, indicating negligible association. Regarding insurance status, 1,235 (23.8%) patients were enrolled in a Self-Pay Discount Program, with 2,538 visits scheduled and 863 (34.0%) no-shows. A total of 874 (16.9%) were enrolled in Medicare, with 1,952 visits scheduled and 479 (24.5%) no-shows. A total of 1,020 (19.7%) were enrolled in Medicaid, with 2,448 visits scheduled and 792 (32.4%) no-shows. A total of 863 (16.7%) were enrolled in Medicare replacement, with 2,058 visits scheduled and 504 (24.5%) no-shows. A total of 151 (2.92%) were enrolled in Medicaid MC Plus, with 305 visits scheduled and 110 (36.1%) no-shows. A total of 622 (12.0%) were enrolled in Blue Cross, with 1,137 visits scheduled and 199 (17.5%) no-shows. Sixty-eight (1.31%) were enrolled in United Healthcare, with 146 visits scheduled and 36 (24.7%) no-shows. Fifty-nine (1.14%) were enrolled in Aetna, with 124 visits scheduled and 31 (25.0%) no-shows. 288 (5.56%) fell into the "other" category, with 571 visits scheduled and 154 (27.0%) no-shows. The differences in no-show rates among these insurance subgroups were statistically significant by chi-squared analysis (chi-squared = 119.26, p<0.001), with Cramer’s V of 0.10, demonstrating a weak association (Table 2).

**Table 2:** No-show rate analysis for 2019, including patient count, visits, no-shows, calculated no-show rate, and statistical significance test results. *Z-statistics were used for comparisons between two groups. **Chi-squared tests were used for categorical variables with more than two groups. n=total number

Variables	Patients (n=5,180)	Visits (n=11,279)	No-shows (n=3,168)	No-show rate (%) (n=28.09)	Test statistic (Z or χ²)	p-Value
Gender						
Male, n (%)	2,501 (48.3)	5,715	1,654	28.94	2.04*	0.04
Female, n (%)	2,679 (51.7)	5,564	1,514	27.21		
Race						
White, n (%)	2,434 (47.0)	5,049	1,195	23.67	63.69**	<0.001
Black, n (%)	2,079 (40.1)	4,932	1,568	31.79		
Other, n (%)	667 (12.9)	1,298	405	31.2		
Ethnicity						
Non-Hispanic, n (%)	4,529 (87.4)	10,057	2,792	27.76	4.21**	>0.05
Hispanic, n (%)	255 (4.92)	519	152	29.3		
Other, n (%)	396 (7.65)	703	224	31.9		
Insurance status						
Self-Pay Discount, n (%)	1,235 (23.8)	2,538	863	34.0	119.26**	<0.001
Medicare, n (%)	874 (16.9)	1,952	479	24.5		
Medicaid, n (%)	1,020 (19.7)	2,448	792	32.4		
Medicare replacement, n (%)	863 (16.7)	2,058	504	24.5		
Medicaid MC Plus, n (%)	151 (2.92)	305	110	36.1		
Blue Cross, n (%)	622 (12.0)	1,137	199	17.5		
United Healthcare, n (%)	68 (1.31)	146	36	24.7		
Aetna, n (%)	59 (1.14)	124	31	25.0		
Other, n (%)	288 (5.56)	571	154	27.0		

In 2020, a total of 4,876 patients were scheduled for 9,990 visits, with 2,606 (26.09%) no-shows. Among them, 48.9% (2,382) were male patients, scheduled for 5,070 visits, with 1,435 (28.30%) no-shows. Female patients made up 51.1% (2,494) of the total, with 4,920 scheduled visits and 1,171 (23.80%) no-shows. The difference between male and female no-show rates was 4.50 percentage points which was statistically significant by two sample z-tests with z-statistic of 5.12 for p<0.001. The effect size as measured by Cohen’s d was 0.103 indicating a small effect size. A total of 2,296 (47.1%) patients were White; this subgroup had 4,586 visits scheduled and 1,069 (23.31%) no-shows. A total of 1,933 (39.6%) patients were Black; this subgroup had 4,202 visits scheduled and 1,221 (29.06%) no-shows. Overall, 647 (13.3%) patients fell into the “other” category; this subgroup had 1,202 visits scheduled and 316 (26.3%) no-shows. The differences in these sub-groups’ no-show rates were statistically significant by chi-squared analysis with a chi-squared value of 27.77 for p<0.001. Cramer’s V was 0.05 demonstrating negligible association. The majority of patients, 4,338 (89.0%), identified as non-Hispanic; this subgroup had 9,018 visits scheduled and 2,346 (26.01%) no-shows. Another 5.99% (292) of patients self-identified as Hispanic, with 565 scheduled visits and 150 (26.5%) no-shows. The remaining 5.05% (246) fell into the "other" category, with 407 scheduled visits and 110 (27.0%) no-shows. The differences in these sub-groups' no-show rates were not statistically significant by chi-squared analysis with a chi-squared value of 0.20 for p>0.05. Cramer’s V was 0.004 demonstrating negligible association.

A total of 1,145 (23.5%) patients were enrolled in a Self-Pay Discount Program; this subgroup had 2,288 visits scheduled and 663 (29.0%) no-shows. Additionally, 663 (13.6%) patients were enrolled in Medicare, with 1,417 scheduled visits and 345 (24.3%) no-shows. Medicaid enrollment included 980 (20.1%) patients, who had 2,248 scheduled visits and 716 (31.9%) no-shows. Among the patients, 867 (17.8%) were enrolled in Medicare replacement, with 1,860 scheduled visits and 464 (24.9%) no-shows. Medicaid MC Plus included 178 (3.65%) patients, with 317 scheduled visits and 100 (31.5%) no-shows. Blue Cross had 625 (12.8%) enrolled patients, with 1,119 visits scheduled and 171 (15.3%) no-shows. For private insurance, 74 (1.52%) patients were enrolled in United Healthcare, with 131 scheduled visits and 23 (17.6%) no-shows, while 70 (1.44%) patients were enrolled in Aetna, with 143 visits scheduled and 25 (17.5%) no-shows. Finally, 274 (5.62%) patients fell into the "other" category, with 467 visits scheduled and 99 (21.2%) no-shows. The differences in no-show rates among insurance subgroups were statistically significant, as determined by chi-squared analysis (χ² = 104.21, p<0.001). Cramer’s V was 0.10, demonstrating a weak association (Table 3). 

**Table 3:** Demographic information of patient populations by year and breakdown of patients by subcategory with percent of total population. *Z-statistics were used for comparisons between two groups. **Chi-squared tests were used for categorical variables with more than two groups. n=total number

Variables	Patients (n=4,876)	Visits (n=9,990)	No-shows (n=2,606)	No-show rate (%) (n=26.09)	Test statistic (Z or χ²)	p-Value
Gender						
Male (%)	2,382 (48.9)	5,070	1,435	28.30	5.12*	<0.001
Female (%)	2,494 (51.1)	4,920	1,171	23.80		
Race						
White (%)	2,296 (47.1)	4,586	1,069	23.31	27.77**	<0.001
Black (%)	1,933 (39.6)	4,202	1,221	29.06		
Other (%)	647 (13.3)	1,202	316	26.3		
Ethnicity						
Non-Hispanic (%)	4,338 (89.0)	9,018	2,346	26.01	0.20**	>0.05
Hispanic (%)	292 (5.99)	565	150	26.5		
Other (%)	246 (5.05)	407	110	27.0		
Insurance status						
Self-Pay Discount (%)	1,145 (23.5)	2,288	663	29.0	104.21**	<0.001
Medicare (%)	663 (13.6)	1,417	345	24.3		
Medicaid (%)	980 (20.1)	2,248	716	31.9		
Medicare replacement (%)	867 (17.8)	1,860	464	24.9		
Medicaid MC Plus (%)	178 (3.65)	317	100	31.5		
Blue Cross (%)	625 (12.8)	1,119	171	15.3		
United Healthcare (%)	74 (1.52)	131	23	17.6		
Aetna (%)	70 (1.44)	143	25	17.5		
Other (%)	274 (5.62)	467	99	21.2		

In 2021, a total of 5,456 patients were scheduled for 11,874 visits, with 3,360 (28.30%) resulting in no-shows. Among them, 47.0% (2,556) were male patients who were scheduled for 5,887 visits and had 1,754 (29.79%) no-shows. Female patients made up 53.0% (2,890) of the total, with 5,987 scheduled visits and 1,606 (26.82%) no-shows. The difference between male and female no-show rates was 2.97 percentage points, which was statistically significant according to a two-sample z-test (z=3.59, p<0.001). The effect size, measured by Cohen’s d, was 0.067, indicating a small effect size. Regarding race, 45.8% (2,497) of patients were White, with 5,357 visits scheduled and 1,305 (24.36%) no-shows. Black patients accounted for 40.7% (2,219) of the total, with 5,060 scheduled visits and 1,671 (33.02%) no-shows. The remaining 13.6% (740) fell into the “other” category, with 1,457 scheduled visits and 384 (26.4%) no-shows. The differences in no-show rates among these racial subgroups were statistically significant, as determined by chi-squared analysis (χ²=71.22, p<0.001). Cramer’s V was 0.08, demonstrating a negligible association. For ethnicity, 89.9% (4,906) of patients identified as non-Hispanic, with 10,791 visits scheduled and 3,055 (28.31%) no-shows. Hispanic patients made up 6.47% (353) of the total, with 727 scheduled visits and 193 (26.5%) no-shows. The remaining 3.61% (197) were categorized as “other,” with 356 scheduled visits and 112 (31.5%) no-shows. The differences in no-show rates among these ethnic subgroups were not statistically significant (χ²=2.05, p>0.05). Cramer’s V was 0.01, indicating a negligible association.

Regarding insurance status, 22.8% (1,244) of patients were enrolled in a Self-Pay Discount Program, with 2,567 scheduled visits and 806 (31.4%) no-shows. Medicare covered 12.3% (672) of patients, with 1,466 scheduled visits and 350 (23.9%) no-shows. Medicaid enrollment included 18.1% (985) of patients, who had 2,467 scheduled visits and 826 (33.5%) no-shows. Another 19.1% (1,044) were enrolled in Medicare replacement, with 2,446 scheduled visits and 666 (27.2%) no-shows. Medicaid MC Plus included 5.54% (302) of patients, with 587 scheduled visits and 219 (37.3%) no-shows. Among private insurance groups, 13.4% (731) of patients were enrolled in Blue Cross, with 1,420 scheduled visits and 269 (18.9%) no-shows. United Healthcare covered 1.80% (98) of patients, with 180 scheduled visits and 28 (15.6%) no-shows. Aetna enrollment included 1.26% (69) of patients, with 135 scheduled visits and 38 (28.1%) no-shows. The remaining 5.70% (311) of patients were classified under the “other” category, with 606 scheduled visits and 158 (26.1%) no-shows. The differences in no-show rates among insurance subgroups were statistically significant, as indicated by chi-squared analysis (χ²=115.41, p<0.001). Cramer’s V was 0.10, demonstrating a weak association (Table 4).

**Table 4:** 2021 no-show rate analysis: patient and visit data, no-show rates, and statistical significance test results. *Z-statistics were used for comparisons between two groups. **Chi-squared tests were used for categorical variables with more than two groups. n=total number

Variables	Patients (n=5,456)	Visits (n=11,874)	No-shows (n=3,360)	No-show rate (%) (n=28.30)	Test statistic (Z or χ²)	p-Value
Gender						
Male (%)	2,566 (47.0)	5,887	1,754	29.79	3.59*	<0.001
Female (%)	2,890 (53.0)	5,987	1,606	26.82		
Race						
White (%)	2,497 (45.8)	5,357	1,305	24.36	71.22**	<0.001
Black (%)	2,219 (40.7)	5,060	1,671	33.02		
Other (%)	740 (13.6)	1,457	384	26.4		
Ethnicity						
Non-Hispanic (%)	4,906 (89.9)	10,791	3,055	28.31	2.05**	0.2
Hispanic (%)	353 (6.47)	727	193	26.5		
Other (%)	197 (3.61)	356	112	31.5		
Insurance status						
Self-Pay Discount (%)	1,244 (22.8)	2,567	806	31.4	115.41**	<0.001
Medicare (%)	672 (12.3)	1,466	350	23.9		
Medicaid (%)	985 (18.1)	2,467	826	33.5		
Medicare replacement (%)	1,044 (19.1)	2,446	666	27.2		
Medicaid MC Plus (%)	302 (5.54)	587	219	37.3		
Blue Cross (%)	731 (13.4)	1,420	269	18.9		
United Healthcare (%)	98 (1.80)	180	28	15.6		
Aetna (%)	69 (1.26)	135	38	28.1		
Other (%)	311 (5.70)	606	158	26.1		

In 2022, a total of 6,017 patients were scheduled for 12,674 visits, with 3,878 (30.60%) resulting in no-shows. Among them, 47.3% (2,847) were male patients who were scheduled for 6,100 visits and had 1,939 (31.79%) no-shows. Female patients made up 52.7% (3,170) of the total, with 6,574 scheduled visits and 1,939 (29.49%) no-shows. The difference between male and female no-show rates was 2.3 percentage points, which was not statistically significant according to a two-sample z-test (z=2.80, p=0.053). The effect size, measured by Cohen’s d, was 0.05, indicating a small effect size. For racial demographics, 45.1% (2,716) of patients were White, with 5,596 scheduled visits and 1,515 (27.07%) no-shows. Black patients accounted for 41.3% (2,484) of the total, with 5,459 scheduled visits and 1,915 (35.08%) no-shows. The remaining 13.6% (817) fell into the “other” category, with 1,619 scheduled visits and 448 (31.2%) no-shows. The differences in no-show rates among these racial subgroups were statistically significant, as determined by chi-squared analysis (χ²=63.09, p<0.001). Cramer’s V was 0.07, demonstrating a negligible association. Regarding ethnicity, 88.4% (5,318) of patients identified as non-Hispanic, with 11,368 visits scheduled and 3,490 (30.70%) no-shows. Hispanic patients made up 7.28% (438) of the total, with 880 scheduled visits and 250 (28.4%) no-shows. The remaining 4.34% (261) were categorized as “other,” with 426 scheduled visits and 138 (32.4%) no-shows. The differences in no-show rates among these ethnic subgroups were not statistically significant (χ²=1.87, p>0.05). Cramer’s V was 0.01, indicating a negligible association.

For insurance status, 12.6% (757) of patients were enrolled in a Self-Pay Discount Program, with 1,246 scheduled visits and 490 (39.3%) no-shows. Medicare covered 9.72% (585) of patients, with 1,217 scheduled visits and 342 (28.1%) no-shows. Medicaid enrollment included 18.4% (1,105) of patients, who had 2,409 scheduled visits and 873 (36.2%) no-shows. Another 21.7% (1,306) were enrolled in Medicare replacement, with 3,132 scheduled visits and 870 (27.8%) no-shows. Medicaid MC Plus included 15.2% (912) of patients, with 2,092 scheduled visits and 689 (32.9%) no-shows. Among private insurance groups, 13.3% (802) of patients were enrolled in Blue Cross, with 1,500 scheduled visits and 333 (22.2%) no-shows. United Healthcare covered 1.98% (119) of patients, with 231 scheduled visits and 49 (21.2%) no-shows. Aetna enrollment included 1.48% (89) of patients, with 174 scheduled visits and 52 (29.9%) no-shows. The remaining 5.68% (342) of patients were classified under the “other” category, with 673 scheduled visits and 180 (26.7%) no-shows. The differences in no-show rates among insurance subgroups were statistically significant, as indicated by chi-squared analysis (χ²=114.94, p<0.001). Cramer’s V was 0.10, demonstrating a weak association (Table 5).

**Table 5:** 2022 no-show rate analysis: the number of patients, visits, and no-shows for each year, no-show rate percentage, and the results of statistical significance tests. *Z-statistics were used for comparisons between two groups. **Chi-squared tests were used for categorical variables with more than two groups. n=total number

Variables	Patients (n=6,017)	Visits (n=12,674)	No-shows (n=3,878)	No-show rate (%) (n=30.60)		p-Value
Gender						
Male (%)	2,847 (47.3)	6,100	1,939	31.79	2.80*	0.053
Female (%)	3,170 (52.7)	6,574	1,939	29.49		
Race						
White (%)	2,716 (45.1)	5,596	1,515	27.07	63.09**	<0.001
Black (%)	2,484 (41.3)	5,459	1,915	35.08		
Other (%)	817 (13.6)	1,619	448	27.7		
Ethnicity						
Non-Hispanic (%)	5,318 (88.4)	11,368	3,490	30.70	1.87**	>0.05
Hispanic (%)	438 (7.28)	880	250	28.4		
Other (%)	261 (4.34)	426	138	32.4		
Insurance status						
Self-Pay Discount (%)	757 (12.6)	1246	490	39.3	114.94**	<0.001
Medicare (%)	585 (9.72)	1,217	342	28.1		
Medicaid (%)	1,105 (18.4)	2,409	873	36.2		
Medicare replacement (%)	1,306 (21.7)	3,132	870	27.8		
Medicaid MC Plus (%)	912 (15.2)	2,092	689	32.9		
Blue Cross (%)	802 (13.3)	1,500	333	22.2		
United Healthcare (%)	119 (1.98)	231	49	21.2		
Aetna (%)	89 (1.48)	174	52	29.9		
Other (%)	342 (5.68)	673	180	26.7		

**Figure 1:**
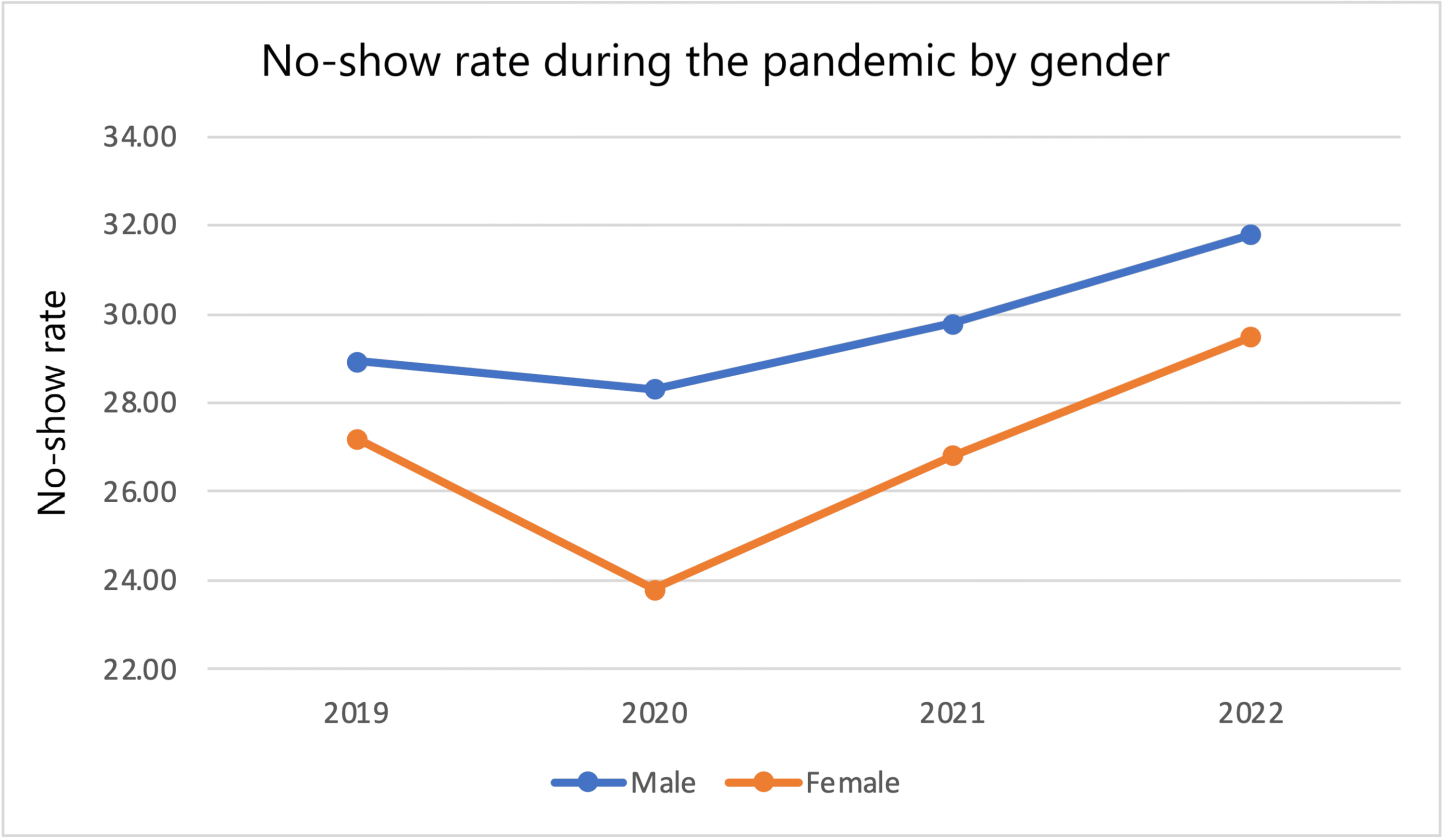
No-show rate during the pandemic by gender.

**Figure 2:**
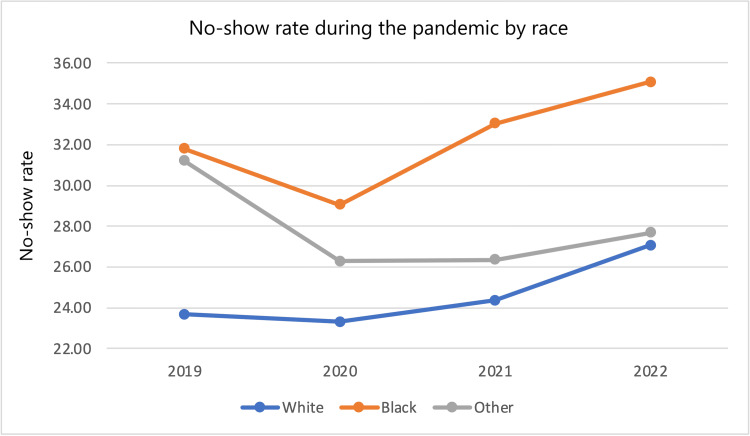
No-show rate during the pandemic by race.

**Figure 3:**
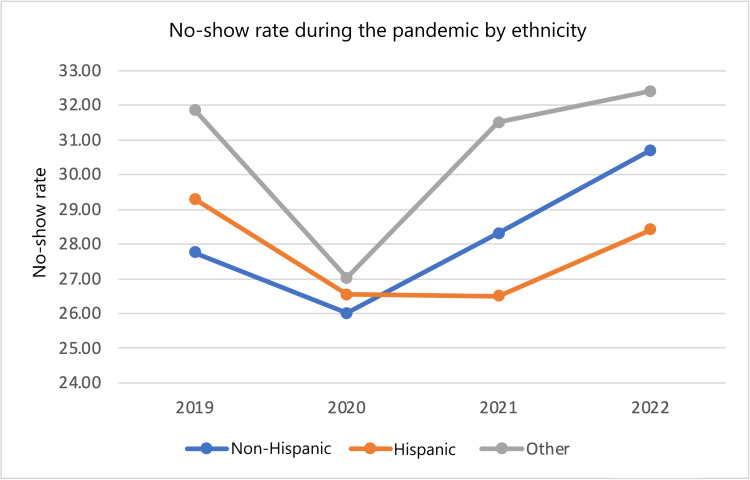
No-show rate during the pandemic by ethnicity.

**Figure 4:**
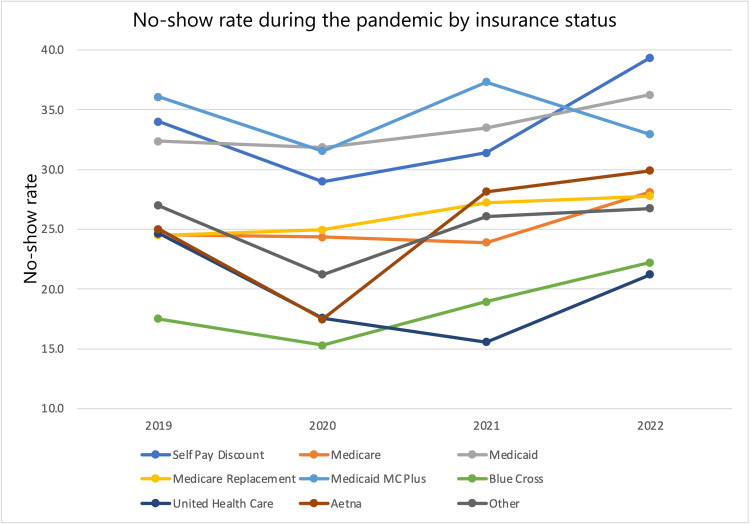
No-show rate during the pandemic by insurance status.

No-show rates among various subgroups from 2022 and 2019 were compared by calculating differences and percent changes in no-show rates. From 2019 to 2022, the entire patient population saw a 2.51 (8.936%) percentage point increase in no-show rate. In that same time frame, male patients saw a 2.85 (9.848%) percentage point increase in no-show rate while female patients saw a 2.28 (8.379%) percentage point increase in no-show rate. White patients saw a 3.40 (14.36%) percentage point increase in no-show rate, Black patients saw a 3.29 (10.35%) percentage point increase in no-show rate, while the “other” category saw a 3.5 (-11.2%) percentage point decrease in no-show rate. Non-Hispanic patients saw a 2.94 (10.59%) percentage point increase in no-show rate, while Hispanic patients saw a 0.9 (-3.07%) percentage point decrease in no-show rate and the “other” category saw a 0.5 (1.57%) percentage point increase in no-show rate. Patients on a Self-Pay Discount plan saw a 5.3 (15.6%) percentage point increase in no-show rate, patients with Medicare saw a 3.6 (14.7%) percentage point increase in no-show rate, patients with Medicaid saw a 3.8 (11.7%) percentage point increase in no-show rate, patients with Medicare replacement saw a 3.3 (13.5%) percentage point increase in no-show rate, patients with Medicaid MC Plus saw a 3.2 (-8.86%) percentage point decrease in no-show rate, patients with Blue Cross saw a 4.7 (26.9%) percentage point increase in no-show rate, patients with United Healthcare saw a 3.5 (14.2%) percentage point decrease in no-show rate, patients with Aetna saw a 4.9 (19.6%) percentage point increase in no-show rate, and patients in the “other” category saw a 0.3 (-1.1%) percentage point decrease in no-show rate. This data in addition to year-by-year percent changes are displayed in Table 6. No-show rates across each year stratified by subgroup are also shown in Figures 1-4.

**Table 6:** Comparison of the no-show rate change from the prior year and between 2022 and 2019.

Variables	2019-2022	2019-2020	2020-2021	2021-2022
Total (difference {percent change})	2.51 (8.936)	-2.00 (-7.12)	2.21 (8.47)	2.30 (8.127)
Gender				
Male (difference {percent change})	2.85 (9.848)	-0.64 (-2.211)	1.49 (5.265)	2.00 (6.714)
Female (difference {percent change})	2.28 (8.379)	-3.41 (-12.53)	3.02 (12.69)	2.67 (9.955)
Race				
White (difference {percent change})	3.40 (14.36)	-0.36 (-1.521)	1.05 (4.505)	2.71 (11.12)
Black (difference {percent change})	3.29 (10.35)	-2.73 (-8.588)	3.96 (13.63)	2.06 (6.239)
Other (difference {percent change})	-3.5 (-11.2)	-4.9 (-15.7)	0.1 (0.38)	1.3 (4.92)
Ethnicity				
Non-Hispanic (difference {percent change})	2.94 (10.59)	-1.75 (-6.304)	2.30 (8.843)	2.39 (8.442)
Hispanic (difference {percent change})	-0.9 (-3.07)	-2.9 (-9.56)	0.0 (0.0)	1.9 (7.17)
Other (difference {percent change})	0.5 (1.57)	-4.9 (-15.4)	4.5 (16.7)	0.9 (2.86)
Insurance status				
Self-Pay Discount (difference {percent change})	5.3 (15.6)	-5.0 (-14.7)	2.4 (8.28)	7.9 (25.2)
Medicare (difference {percent change})	3.6 (14.7)	-0.2 (-0.816)	-0.4 (-1.65)	4.2 (17.6)
Medicaid (difference {percent change})	3.8 (11.7)	-0.5 (-1.54)	1.6 (5.02)	2.7 (8.06)
Medicare replacement (difference {percent change})	3.3 (13.5)	0.4 (1.63)	2.3 (9.24)	0.6 (2.21)
Medicaid MC Plus (difference {percent change})	-3.2 (-8.86)	-4.6 (-12.7)	5.8 (18.4)	-4.4 (-11.8)
Blue Cross (difference {percent change})	4.7 (26.9)	-2.2 (-12.6)	3.6 (23.5)	3.3 (17.5)
United Healthcare (difference {percent change})	-3.5 (-14.2)	-7.1 (-28.7)	-2.0 (-11.4)	5.6 (35.9)
Aetna (difference {percent change})	4.9 (19.6)	-7.5 (-30.0)	10.6 (60.6)	1.8 (6.41)
Other (difference {percent change})	-0.3 (-1.1)	-5.8 (-21.5)	4.9 (23.1)	0.6 (2.3)

## Discussion

Our study sought to investigate the effects of the pandemic on access to outpatient cardiovascular care in a safety-net hospital system to determine how patients of low socioeconomic status were affected. To do this we analyzed no-show rates at outpatient cardiology clinics within the University Health System by different patient demographics. Our data were collected in 2023 and spans from pre-pandemic in 2019 to 2022. Although the pandemic was not over in 2022, the most drastic policies affecting daily life, like lockdowns, had subsided such that most people’s activities more closely resembled pre-pandemic patterns. The present study found that the majority of patient groups in our study saw an increase in no-show rates over the full-time period observed. When all patients were included, a 2.51 (8.936%) percentage point increase in no-show rate was observed. Both male and female subgroups saw similar percent increases in no-show rates. Among racial subgroups, both White and Black patient subgroups accounting for 11,125 (85.74%) patients saw a percent increase in no-show rates. Similarly, non-Hispanic patients and those who fell into the “other” category, making up 12,103 (93.27%) patients saw an increase in no-show rates. Excluding Medicaid MC Plus, United Healthcare, and those in the “other” group, all other insurance subgroups making up 7,770 (59.88%) patients saw an increase in no-show rates.

One interesting caveat in the global trend is that no-show rates decreased in all patient subgroups except Medicare Replacement from 2019 to 2020. However, there was also a steep decrease in scheduled visits from 11,279 in 2019 to 9,990 in 2020 that normalized with 11,874 patient visits in 2021. This global decrease in no-show rates likely reflects a trend in prioritization of patient visits in the outpatient setting rather than an increase in patient attendance. As noted by Yasmin et al., many societies enacted policies in the early pandemic calling for prioritization of more urgent and emergent cases and evaluations rather than the full spectrum of treatment in non-COVID-19 patients [1]. We suspect that the patients who were scheduled for outpatient visits in 2020 were more carefully selected based on the urgency of care needs and thus had higher motivation to attend visits.

The effects of the pandemic were felt by many, but some subgroups suffered more. Data were first stratified by gender. Male patients had higher no-show rates and percent increases in no-show rates compared to their female counterparts. Men had higher no-show rates in all years examined, and the difference was statistically significant in all years compared except 2022. Data were also stratified by Race. Black and White patients both saw an increase in no-show rates. White patients saw a larger percent change in no-show rates (3.40 {14.36%} compared to 3.29 {10.35%}). However, Black patients had a greater baseline and final no-show rate during the study period (1,568 {31.79%} and 1,915 {35.08%}, respectively). The latter number is specifically concerning as it suggests that more than one-third of Black patients did not receive the cardiovascular care they needed. The differences in racial subgroups were statistically significant in every year observed. Data were also stratified by ethnicity. In this group of stratification, patients who identified as “non-Hispanic” represented the largest proportion of patients with 11,427 total patients over all four years. Given that this group represented a much larger proportion than the other groups (11,427 {88.06%} patients compared to 873 {6.73%} Hispanic patients and 676 {5.21%} “other” patients), it makes sense that their no-show rate trend mirrors that of the global trend in increased no-show rate at 2.94 (10.95%) more percentage points compared to 2.51 (8.936%) more percentage points for the total population. In contrast to the global trend, within the ethnicity strata, the Hispanic group saw a 0.9 (3.07%) percentage point decrease in no-show rate. This may be secondary to a 183 (71.76%) patient increase in population (255 in 2019 to 438 in 2022) resulting in 152 no-shows in 2019 creating a bigger effect than 250 no-shows in 2022, but the cause is unclear. The final stratification was insurance status. In this subset, nearly every group saw a 10-16% increase in no-show rates with exceptions being “Medicaid MC Plus” (3.2 {8.86%} percentage point decrease), “Blue Cross” (4.7 {26.9%} percentage point increase), “United Healthcare” (3.5 {14.2%} percentage point decrease), “Aetna” (4.9 {19.6%} percentage point increase), and the “other” category (0.3 {-1.1%} percentage point decrease).

Overall, 12/17 (71.0%) subgroups saw an increase in no-show rates with seven groups having no-show rates of 30% or greater in 2022 - male patients, Black patients, non-Hispanic patients, patients in the “other” category of ethnicity, those on a Self-Pay Discount Program, Medicaid, Medicaid MC Plus. These groups all had no-show rates suggesting that nearly or greater than a third of their patients were not receiving the cardiovascular care they needed by the end of the pandemic. This is a particularly troublesome finding in the Self-Pay Discount patients (863 {39.3%} no-shows), Medicaid (1,020 {36.2%} no-shows), and Medicaid MC Plus (110 {32.9%} no-shows), as these insurance groups serve as our best markers of low socioeconomic status. University Health System is a safety net hospital that offers a wide spectrum of resources for low socioeconomic status patients. This includes transportation services to facilitate appointments, free low-cost medications and healthcare services, and many other areas like assistance with financial planning and housing. Even in a safety-net hospital system like this one with copious resources to facilitate healthcare access for the patients who need it, the effects of the pandemic in exacerbating the detrimental effects of social determinants of health were clear.

A similar study examining no-show rates during 2020 among 72,000 patients found that males were more likely to no-show than females during in-person visits (OR: 1.15, 95% CI: 1.08-1.22, p<0.01) [7]. This study collected data from 2020, where we also saw the highest difference in no-show rates between genders (1,435 {28.30%} for males and 1,171 {23.80%} for females). As shown in Figure 1, after 2020 this gap began to decrease and more closely resembled the difference in 2019. In the same study, Black race compared to White race was associated with higher no-show rates (OR: 1.36, 95% CI: 1.24-1.48, p<0.01) as was Medicaid (OR: 1.23, 95% CI: 1.13-1.35, p<0.01) and Medicare (OR: 1.11, 95% CI: 1.01-1.22, p=0.04) compared to commercial insurance [7]. However, their study also compared no-show rates for telemedicine visits to in-person visits and found that sociodemographic factors associated with higher odds ratios for no-showing had less effect on telemedicine visits [7]. A very large study collecting similar data from 2021 to 2022 from nearly two million outpatient encounters across nine specialties similarly found that Black race, Medicaid, and Medicare were associated with higher odds of no-showing but that these effects were reduced in telehealth visits [8]. Many studies have documented decreases in outpatient visits in cardiology clinics during the pandemic [4,5,9,10]. However, clinics that employed the use of telehealth visits saw a near comparative compensation with one study reporting after initially seeing visit volumes below pre-COVID levels in March-May of 2020; they saw visit volumes exceed pre-COVID levels after June 2020 [9,10]. That same study by Kalwani et al. noted that from June 2020 to February 2021 subspecialties delivering a greater percentage of visits through telemedicine saw larger increases in new patient visits. Some may be concerned that telehealth is not a sufficient substitute, particularly in conditions like heart failure where physical exam is particularly relevant to patient assessment. One investigation examining 8,263 patients with heart failure, compared approximately 4,500 propensity-matched patients whose visits were conducted by telehealth in 2020 to 4,500 patients with in-person visits in the same year and found no significant difference in mortality, excess in hospital encounters, or need for intensive care [11].

The COVID-19 pandemic placed significant strain on the healthcare system and exacerbated disparities in healthcare access in certain sociodemographic groups and as shown in our study, those of low socioeconomic status were affected the most. The pandemic forced the entire healthcare system into an experiment advancing telemedicine. As described in the studies above, telemedicine helped ameliorate the effects of certain sociodemographic factors in reducing access to healthcare. Further research is needed into the quality and safety of telemedicine as a substitute for inpatient visits, and into the ability of telemedicine to truly achieve the effects noted, but this experiment with telemedicine during the pandemic may have provided us with the key to bridging healthcare access gaps in patients of low socioeconomic status.

Limitations of this study include restrictions in the data we were able to collect about missed appointments and patient demographics. Although we were able to identify no-shows, we were unable to determine whether the appointment was later made up or what circumstances led to the missed appointment. This includes the factors that led clinic staff to mark visits as no-shows. Unfortunately, this may have varied between clinics and even among different staff members. We felt that the no-show rate represented the best surrogate measure of care that was missed due to the effects of the pandemic but recognize that it is an imperfect measure. We elected to exclude rescheduled and canceled visits, as these introduced the effect of the provider’s schedule, but we acknowledge that rescheduled visits may also have been influenced by the pandemic. Future studies should investigate these types of visits as well.

Additionally, we were unable to analyze the number of patients lost to follow-up or new to the clinic and, therefore, could not assess changes in clinic retention and patient influx. Our “other” category, while representing some smaller racial subgroups such as Asian and Native American populations, was primarily composed of entries in the electronic medical record (EMR) such as “patient declined to answer,” “multiple,” “other,” “unspecified,” “unavailable,” or simply blank responses. We included this category in the analysis since it technically represents all patients outside the specified subgroups; however, due to the inclusion of these miscellaneous EMR entries, it remains highly heterogeneous and variable. One reason for this variability is that patients who fell into this category at one visit due to a lack of entry or an alternative entry may have correctly categorized themselves in other visits. When each group was analyzed independently, the patient count varied significantly year by year, leading to the decision to combine the smaller groups into a single “other” category.

Finally, one might argue that the peak of the COVID-19 pandemic spanned from 2020 through 2024. Since our data were collected in 2023, we unfortunately do not have complete data for the entire period. However, most published studies similar to ours only included data up to 2022 at the latest, and many contained only one year’s worth of data, typically from 2020 or 2021. Although the pandemic continued to have flare-ups into 2024, we believe 2022 marks a significant milestone, as most aspects of daily life returned to normal, with major restrictions and policy-driven impacts on social determinants of health (SDOH), such as transportation access, largely coming to an end.

## Conclusions

Our study analyzed no-show rates at outpatient cardiology clinics within a safety-net hospital system to determine the effects of the pandemic on outpatient cardiovascular care of low socioeconomic status patients. We found that 12 out of 17 (71.0%) patient subgroups analyzed saw an increase in no-show rates from 2019 to 2022, with seven subgroups exhibiting no-show rates near or above one-third of total clinic visits (male patients, Black patients, non-Hispanic patients, patients in the “Other” category of ethnicity, those on a Self-Pay Discount Program, Medicaid, Medicaid MC Plus). Even in a safety-net hospital system like University Health in Kansas City, MO, that offers a myriad of resources to facilitate healthcare access for the patients who need it, the detrimental effects of the pandemic on outpatient cardiovascular care access were clear.
